# Computational Prediction of Candidate Proteins for S-Nitrosylation in *Arabidopsis thaliana*

**DOI:** 10.1371/journal.pone.0110232

**Published:** 2014-10-21

**Authors:** Mounira Chaki, Izabella Kovacs, Manuel Spannagl, Christian Lindermayr

**Affiliations:** 1 Institute of Biochemical Plant Pathology, Helmholtz Zentrum München-German Research Center for Environmental Health, Neuherberg, Germany; 2 Institute of Bioinformatics and Systems Biology, Helmholtz Zentrum München-German Research Center for Environmental Health, Neuherberg, Germany; Huazhong University of Science and Technology, China

## Abstract

Nitric oxide (NO) is an important signaling molecule that regulates many physiological processes in plants. One of the most important regulatory mechanisms of NO is S-nitrosylation—the covalent attachment of NO to cysteine residues. Although the involvement of cysteine S-nitrosylation in the regulation of protein functions is well established, its substrate specificity remains unknown. Identification of candidates for S-nitrosylation and their target cysteine residues is fundamental for studying the molecular mechanisms and regulatory roles of S-nitrosylation in plants. Several experimental methods that are based on the biotin switch have been developed to identify target proteins for S-nitrosylation. However, these methods have their limits. Thus, computational methods are attracting considerable attention for the identification of modification sites in proteins. Using GPS-SNO version 1.0, a recently developed S-nitrosylation site-prediction program, a set of 16,610 candidate proteins for S-nitrosylation containing 31,900 S-nitrosylation sites was isolated from the entire *Arabidopsis* proteome using the medium threshold. In the compartments “chloroplast,” “CUL4-RING ubiquitin ligase complex,” and “membrane” more than 70% of the proteins were identified as candidates for S-nitrosylation. The high number of identified candidates in the proteome reflects the importance of redox signaling in these compartments. An analysis of the functional distribution of the predicted candidates showed that proteins involved in signaling processes exhibited the highest prediction rate. In a set of 46 proteins, where 53 putative S-nitrosylation sites were already experimentally determined, the GPS-SNO program predicted 60 S-nitrosylation sites, but only 11 overlap with the results of the experimental approach. In general, a computer-assisted method for the prediction of targets for S-nitrosylation is a very good tool; however, further development, such as including the three dimensional structure of proteins in such analyses, would improve the identification of S-nitrosylation sites.

## Introduction

NO is a membrane-permeable free radical that plays a central role in a broad spectrum of physiological processes in plants, including germination, flowering, root development, hormonal signaling, senescence, and the establishment of adaptive responses against biotic and abiotic stress [1]–[9]. NO and related nitrogen species that are considered reactive can mediate various post-translational modifications (PTMs), such as metal nitrosylation, tyrosine nitration, and cysteine S-nitrosylation. Cysteine S-nitrosylation is the term used to describe the covalent binding of an NO group to a protein cysteine (Cys) residue. This PTM is considered one of the most important molecular mechanisms by which NO regulates protein functions and cell signaling and has been shown to alter protein activities, protein-protein interactions, and subcellular localization under both normal and pathological conditions [10]–[13].

A number of indirect MS-based proteomics approaches have been developed for the identification of S-nitrosylated proteins and their modification sites from complex biological samples [14], [15]. The biotin switch technique (BST) is the most widely used method and is based on the conversion of S-nitrosylated Cys to biotinylated Cys. Such labeling allows the detection of S-nitrosylated proteins using specific anti-biotin antibodies and their isolation by affinity chromatography using neutravidin matrices. The proteins can then be identified using mass spectrometry. S-nitrosoglutathione (GSNO) is the most abundant low-molecular-weight S-nitrosothiol in plant cells and is a physiological NO reservoir and NO donor. This molecule can transfer its NO moiety to protein cysteine residues via trans-nitrosylation. GSNO has often been used to generate S-nitrosylated proteins in extracts for the subsequent isolation and identification of S-nitrosylated proteins [16]–[20].

The identification of redox-sensitive cysteine residues is important for understanding the regulatory functions of NO. Cysteine residues exhibiting a low-pKa sulfhydryl group are particularly susceptible to certain types of redox modification [21]. Several research groups have attempted to define consensus motifs for S-nitrosylation by comparing the amino acid sequences around identified target cysteine residues. Such analyses have revealed that the target cysteine residues often lie within an acid-base or hydrophobic motif [22]. In contrast, other studies have revealed that the primary sequence of the surrounding amino acid residues has no significant effect on the reactivity of cysteines towards S-nitrosylation at the peptide level [23]. Greco et al. (2006) supported the idea of extending the motif beyond the primary sequence to include hydrophobic motifs surrounding the identified cysteine residues [24]. Recently, 70 known S-nitrosylated sites were used to identify general structures associated with S-nitrosylation. The results obtained revealed that proximal acid–base motif, Cys pKa, sulfur atom exposure, and Cys conservation or hydrophobicity in the vicinity of the modified cysteine do not predict S-nitrosylation specificity. Instead, this analysis identified a revised acid-base motif that is located farther from the cysteine and in which the charged groups are exposed [25].

Many studies have been performed to identify and characterize S-nitrosylated proteins in plants [26]. The pioneer analysis of S-nitrosylated proteins was conducted in 2005 [16]. In this work, 63 proteins from GSNO-treated *Arabidopsis* cell culture extracts and 52 proteins from NO-treated leaves were identified as possible NO targets. In addition, Romero-Puertas and colleagues found 16 *Arabidopsis* proteins that were differentially S-nitrosylated under hypersensitive responses [27]. Moreover, endogenous S-nitrosylated proteins have been identified in an *Arabidopsis* cell culture under salt stress [28]. To date, more than two hundred proteins have been identified as putative targets for S-nitrosylation in *Arabidopsis* using proteomics approaches based on the biotin switch assay or related techniques, however only in the minority of them the exact S-nitrosylation sites have been identified. Moreover, such analyses have also been performed in other plant species such as in citrus plants exposed to salinity [29], a rice mutant overproducing NO [30], pea-leaf peroxisomes under abiotic stress [31], and a tobacco cell suspension treated with cryptogein [32]. The S-nitrosylated proteins identified from plant proteome studies have been shown to participate in major cellular activities, notably primary and secondary metabolism, protein folding and genetic information processing, photosynthesis, cellular architecture, and responses to biotic and abiotic stresses [33]. Although the number of plant proteins that have been identified as putative targets for S-nitrosylation has drastically increased during recent years, studies identifying the NO-sensitive cysteine residues involved remain rare. These analyses are essential for a better understanding of the function of protein S-nitrosylation in plants [33].

In contrast to the technical difficulties associated with experimental methods, the computational analysis of PTMs is an attractive alternative. The use of computational predictors can identify a number of potential candidates and rapidly generate useful information. Currently, approximately 170 databases and computational tools have been developed for PTM analysis [34]. The algorithms used in this field include iGPS 1.0, which is used to predict phosphorylation [35], CSS-Palm 4.0, which is used to predict S-palmitoylation [36], GPS-SUMO 1.0, which is used to predict sumoylation [37], and GPS-YNO2, which is used to predict protein nitration [38]. Moreover, several programs and algorithms have been developed to predict cysteine residues that are susceptible to S-nitrosylation, including SNOSite, iSNO-PseAAC, iSNO-AAPair, and GPS-SNO 1.0 [39]–[42].

In this study, we used GPS-SNO 1.0 to identify candidate proteins for S-nitrosylation within the *Arabidopsis* proteome (27,416 proteins). In total, 31,907 S-nitrosylated sites were predicted in 16,610 (approximately 61%) candidate proteins using the medium threshold. Potential target proteins were detected in all cellular compartments and ranged from 37% to 86% of the total number of proteins per compartment. More than 70% of the S-nitrosylated candidates identified were in the “chloroplast”, “CUL4-RING ubiquitin ligase complex”, and “membrane” compartments. In most compartments, the proportion of S-nitrosylation candidates was approximately 60%. Moreover, the 10% of S-nitrosylation sites with the highest prediction confidence were extracted for further study. This group comprised 3,190 sites in 3,005 target proteins. These candidates were detected in all compartments and ranged from 5% to 17% of the total number of proteins per compartment. These targets were enriched in the “chloroplast” (17%), “intracellular” (15%), and “plasmodesmata” (14%) compartments. In most compartments, the percentage of proteins predicted as S-nitrosylation candidates was approximately 10%. The high proportion of proteins identified as S-nitrosylation candidates reflects the importance of redox signaling in these compartments. An analysis of the functional distribution of the predicted candidates showed that the group with the highest prediction rate was the process “signaling”. Moreover, a set of 46 *Arabidopsis* proteins, where 53 putative S-nitrosylation sites were previously determined using a BST-based approach, was analysed with the GPS-SNO program. The computational method predicted 60 S-nitrosylation sites within these proteins, but only 11 overlap with the results of the BST-based approach. In general, the currently available algorithm appears to be a useful tool for characterizing the S-nitrosylome but requires further improvement regarding its accuracy in identifying S-nitrosylation sites.

## Materials and Methods

### Data collection

First, 27,416 amino acid sequences were downloaded from the most recent version of the *Arabidopsis* information resource TAIR (TAIR10, www.arabidopsis.org). For all subsequent analyses, only one representative gene model was used per locus.

### Comparison of prediction performance

To evaluate and compare the prediction performance of four S-nitrosylation prediction programs, we calculated 3 parameters (accuracy, sensitivity, and specificity) according to the definitions described previously [42]. The 4 prediction programs tested were GPS-SNO 1.0 (using the medium threshold condition), iSNO-PseAAC, iSNO-AAPair, and SNOSite [39]-[41].

### Prediction of SNO sites using GPS-SNO software

Group-based Prediction System (GPS-SNO 1.0) software was used to predict S-nitrosylation sites [42]; this program can be executed online or downloaded at http://sno.biocuckoo.org/. In all analyses, 27,416 *Arabidopsis* amino acid sequences in FASTA format were submitted for use in predicting S-nitrosylation sites under the medium threshold condition using the batch prediction tool of the GPS-SNO 1.0 software. The predicted S-nitrosylation sites were extracted into an Excel file for further analysis.

### Subcellular compartmentalization of *Arabidopsis* proteins

To determine the cellular localization of all gene predictions in *Arabidopsis*, we utilized gene ontology terms (GO) obtained from the TAIR10 annotation release (ftp://ftp.arabidopsis.org/home/tair/Ontologies/Gene_Ontology/) and filtered these terms for terms categorized as “cellular component”. The distribution of proteins among the individual localization categories was plotted for all categories comprising more than 100 assignments.

### MapMan analysis of the predicted candidate proteins

Protein functional classification was performed according to the MapMan Ontology of *Arabidopsis* proteins, version 3.5.1R2 (http://mapman.gabipd.org/web/guest/mapman).

## Results and Discussion

In recent years, many experimental methods have been developed for the identification of S-nitrosylated proteins and the mapping of SNO-sites. The BST and related methods have enabled the high-throughput identification of hundreds of novel targets for S-nitrosylation [16], [18], [43]-[45]. However, these methods have several limitations, especially regarding the detection of low-abundance or unstable proteins or of proteins that are present only in specific tissues/organs that are difficult to handle, e.g., meristems or epidermis. Therefore, more sensitive approaches are required. ProteoMiner is a technology allowing the enrichment of low-abundance proteins [46]. However, the extracted proteins are denatured by the harsh conditions required for protein elution. Therefore, this method cannot be used in combination with the BST until a method for enriching low-abundance proteins under native conditions is established. Computational methods can overcome such technical difficulties because the analyses can be performed using the complete protein datasets that are available in databases. Thus, a nearly complete map of candidates for S-nitrosylation can be generated, providing a good starting point for more detailed, experimental approaches.

### 1. A comparison of programs used to predict S-nitrosylation sites

Previously, we compared three programs that are used to predict S-nitrosylation sites in proteins [26]. Here, we extended this study by including a fourth program and including all plant proteins in which modified cysteine residues have been verified using mass spectrometry and for which the physiological functions are known (Table 1). The programs GPS-SNO 1.0, iSNO-PseAAC, iSNO-AAPair, and SNOSite were tested. The performances of the 4 programs in predicting S-nitrosylation were evaluated (Table S1) as previously defined [42], using the 12 characterized S-nitrosylated proteins listed in Table 1. GPS-SNO performed best according to the three criteria chosen (accuracy, sensitivity, and specificity; 82.2%, 50%, and 87.9%, respectively, Table S1). The SNOSite software predicted almost all cysteine residues present as targets for S-nitrosylation, with accuracy and specificity of 25% and 13%, respectively, which implies that S-nitrosylation is very unspecific. The programs iSNO-PseAAC and iSNO-AAPair presented higher accuracy and specificity than SNOSite (Table S1), but their correlation with actual sites remained low. Significantly better predictions appeared possible when using the GPS-SNO 1.0 software, which exhibited a much lower rate of false positives. Approximately 60% of the proteins that were found to be S-nitrosylated using mass spectrometry were predicted using the GPS-SNO 1.0 software (which was developed by Xue and colleagues [42]). The authors of this program have improved their previous algorithm, GPS 2.0 (Group-based Prediction System), which was used for the prediction of kinase-specific phosphorylation sites, and have released GPS 3.0 [47]. Based on this algorithm, they developed the computational software GPS-SNO 1.0 for the prediction of S-nitrosylation sites. The performance of the GPS 3.0 algorithm at predicting S-nitrosylation was much better than that obtained using several other approaches, providing an accuracy of 75.70%, a sensitivity of 53.32% and a specificity of 80.11% under the low threshold condition. GPS-SNO 1.0 was applied to a test set of 485 potentially S-nitrosylated proteins collected from PubMed. These proteins were identified in large- or small-scale studies, and the actual S-nitrosylation sites have not been experimentally determined. Of the analyzed proteins, 371 (approximately 76%) were predicted to be S-nitrosylated at one or more potential S-nitrosylation sites.

**Table 1:** Computational prediction of *S*-nitrosylation sites from experimentally identified *S*-nitrosylated proteins in plants using GPS-SNO 1.0, iSNO-PseAAC, iSNO-AAPair, and SNOSite software.

Protein name	Accession number	Total number of Cys	Physiological function demonstrated	Cys-NO sites identified by LC-MS/MS	Cys-NO sites predicted by GPS-SNO 1.0	Cys-NO sites predicted by iSNO-PseAAC	Cys-NO sites predicted by iSNO-AAPair	Cys-NO sites predicted by SNOSite	Reference
Methionine adenosyltransferase 1	At1g02500	8	Inhibited	C_114_	C_114_	C_161_	C_31_, C_90_, C_161_	C_20_, C_31_, C_42_, C_73_, C_90,_ C_114_, C_161_	[85]
Metacaspase 9	At5g04200	7	Inhibited	C_147_	C_17_, C_147_	C_17_, C_29_	C_117_	C_17_, C_29_, C_117_, C_147_, C_309_	[81]
Peroxiredoxin II E	At3g52960	2	Inhibited	C_121_	C_121_	C_121_, C_146_	C_121_	C_121_, C_146_	[86]
NPR1	At1g64280	17	Inhibited	C_156_	C_156_, C_385_	C_212_, C_306_	C_223_, C_306_, C_394_,C_457_	C_82_, C_150_, C_155_, C_156_, C_160_, C_212_, C_223_, C_297_, C_306_, C_378_, C_385_, C_394_, C_457_, C_511_, C_529_	[83]
GAPDH	At1g13440	2	Inhibited	C_156_, C_160_	C_156_, C_160_	_	_	C_156_, C_160_	[87]
SABP3	At3g01500	7	Inhibited	C_280_	C_34_, C_173_, C_280_	C_230_, C_257_	C_34_	C_34_, C_167_, C_173_, C_230_, C_257_, C_277_, C_280_	[88]
Transcription factor-TGA1	At5g65210	4	Activated	C_172_, C_287_	C_172_	_	_	C_172_, C_260_, C_266_, C_287_	[19]
NADPH oxidase	At5g47910	10	Inhibited	C_890_	_	C_208_, C_387_, C_433_, C_480_, C_695_	C_412_, C_480_, C_695_, C_890_	C_208_, C_410_, C_412_, C_433_, C_480_, C_651_, C_695_, C_825_, C_890_	[89]
cALD2	At2g36460	6	Inhibited	C_173_	C_68_, C_326_	C_326_	C_208_	C_68_, C_173_, C_197_, C_208_, C_326_	[90]
TIR1	At3g62980	23	Activated	C_140_	C_516_, C_551_	C_34_, C_53_, C_121_, C_140_, C_155_, C_210_, C_269_, C_288_, C_311_, C_405_, C_480_, C_491_	C_121_, C_140_, C_405_, C_551_	C_34_, C_44_, C_53_, C_121_, C_140_, C_155_, C_193_, C_210_, C_264_, C_269_, C_288_, C_311_, C_337_, C_371_, C_405_, C_480_, C_491_, C_516_, C_523_, C_551_	[91]
CDC48	Q1G0Z1	14	Inhibited	C_110_, C_526_, C_664_	C_426_, C_576_	C_74_, C_82_, C_110_, C_526_, C_539_, C_576_, C_664_, C_699_	C_74_, C_426_, C_539_, C_576_	C_74_, C_82_, C_110_, C_179_, C_189_, C_272_, C_419_, C_426_, C_539_, C_576_, C_664_, C_695_, C_699_	[32]
AtMYB30	At3g28910	7	Inhibited	C_53_	C_6_	C_6_, C_7,_ C_49_, C_53_, C_257_, C_289_	C_6_, C_7_	C_49_, C_53_, C_257_, C_289_, C_290_	[92]

Amino acid sequences were downloaded from the most recent version of the *Arabidopsis* information resource TAIR (TAIR10, www.arabidopsis.org) and subjected to the different programs for prediction of S-nitrosylation sites. NPR1, non-expresser of pathogenesis related genes 1; GAPDH, glyceraldehyde 3-phosphate dehydrogenase; SABP3, salicylic acid binding protein 3; TGA1, TGACG motif binding factor; cALD2, cytosolic fructose 1,6-bisphosphate aldolase; TIR1, transport inhibitor response 1; CDC48, cell division cycle 48; AtMYB30, *Arabidopsis thaliana* MYB transcription factor.

C in bold, matched cysteine residues, "_" not predicted

### 2. Prediction of S-nitrosylation candidate proteins using the GPS-SNO 1.0 program

For the computer-based prediction of the S-nitrosylation of *Arabidopsis* target proteins, 27,416 amino acid sequences were extracted from the TAIR 10 database (www.arabidopsis.org) (Table S2). Of these proteins, 25,785 (94%) contain at least one cysteine residue; in total, 207,473 cysteine residues were found. All of the *Arabidopsis* amino acid sequences were analyzed with GPS-SNO 1.0 using the medium threshold, as recommended by Xue and colleagues [42]. In total, 31,907 (approximately 15% of all Cys residues) S-nitrosylation sites were predicted in 16,610 proteins (60%) (Table 2 and Table S2 and S3), suggesting that redox-related processes are closely regulated by a small number of redox-sensitive cysteine residues. The high number of putative candidate proteins reflects the importance of redox-signaling in general. Redox homeostasis during development is an evolutionary conserved strategy and the common origin of redox sensing indicate that organisms evolved similar strategies for utilizing redox-signaling during development [48]. In plant with impaired NO/S-nitrosothiol (SNO) homeostasis the importance of balancing NO/SNO levels for plant growth and development become apparent. For instance, S-nitrosoglutathione reductase knock-out plants have higher SNO levels in comparison to wild type plants and display a lot of different developmental defects, such as delayed seed germination, reduced growth, reduced trichome density, increased number of branched shoots, and generation of more flowers, which are smaller and develop to smaller siliques containing smaller seeds [49]. Moreover, leaf shape, 2,4-D sensitivity, and hypocotyl elongation is affected [50]. But S-nitrosylation of proteins might have not only a signaling function. A protection of cysteine residues against irreversible oxidation is also described [51], [52]. In this way proteins can be protected against oxidative damage and after reduction they can fulfil their physiological function again.

**Table 2:** Prediction of *Arabidopsis* candidate proteins for S-nitrosylation using the GPS-SNO 1.0 software.

	*Arabidopsis* proteome	Candidate proteins for *S*-nitrosylation	The highest 10% high-confident predicted candidates
Total number of proteins	27,416	16,610 (60%)	3,005 (18%)
Total number of Cys-NO	207,473	31,907 (15%)	3,190 (10%)

*Arabidopsis* amino acid sequences were extracted from TAIR 10 database (www.arabidopsis.org) and analysed by GPS-SNO 1.0 software using medium threshold condition. The 10% of predicted sites with the highest prediction confidence were determined by ranking the prediction results according to the raw score divided by the threshold (Cutoff) for a particular cluster.

On the other side, the high number of putative candidate proteins might indicate a high rate of false-positive predictions. Therefore, we extracted the 10% of predicted sites with the highest prediction confidence by ranking the prediction results according to the raw score divided by the threshold (Cutoff) for a particular cluster. These sites (3,190) were localized to 3005 different proteins, which comprise 18% of all predicted S-nitrosylation candidates (Table 2 and Table S2 and S3). Similarly, computational prediction has also been used for other post-translational modifications of target proteins. In the *Arabidopsis* proteome, the phosphorylation hotspot prediction algorithm has predicted 13,677 P-hotspots in 9,599 proteins corresponding to 7,847 unique genes [53]. The cited study provides a new bioinformatic method to identify phosphorylation hotspots and provides the basis for further investigation of novel candidate P-hotspots. Moreover, in the human proteome, nitration-sensitive tyrosine residues have been predicted using GPS-YNO2, a recently described 3-nitrotyrosine prediction algorithm [54]. In total, 9.27% (27,977) of all tyrosine residues (301,091) were predicted to be nitration targets. Collectively, these studies demonstrate the feasibility of using predicted datasets for whole-proteome analyses.

### 3. Subcellular compartment classification of *Arabidopsis* proteins

To determine whether the identified candidates for S-nitrosylation are enriched in distinct subcellular compartments (Text S1), all *Arabidopsis* proteins and the predicted candidates were assigned to subcellular locations according to gene ontology (GO) terms using cellular component classifications (Table S4). In Table 3, only compartments with more than 100 representatives are listed. An analysis of the subcellular localization of all *Arabidopsis* proteins revealed that most were assigned to the “nucleus” (9,214 proteins) or to “membranes” (4,389 proteins). The predicted S-nitrosylation candidate proteins were also located in other compartments, comprising 37% to 86% of the total protein content in each compartment (Table 3). Similar results have been found experimentally in *Arabidopsis* suspension cell cultures: S-nitrosylated proteins were found in almost all cell compartments [28]. Moreover, a similar distribution was also observed in animal cells [55]. Interestingly, the predicted candidates are most enriched in the “chloroplast”, “CUL4-RING ubiquitin ligase complex”, and “membrane” compartments (86%, 75%, and 74%, respectively), suggesting that redox-related processes play important roles in these locations.

**Table 3:** Subcellular compartment classification of *Arabidopsis* proteins.

Compartments	Total number of proteins	Candidate proteins for *S*-nitrosylation	Candidate proteins for *S*-nitrosylation harboring the highest 10% high-confident predicted sites
Chloroplast	3795	3259 (86%)	659 (17%)
CUL4-RING ubiquitin ligase complex	121	91 (75%)	13 (11%)
Membrane	4389	3257 (74%)	493 (11%)
Plasmodesmata	848	596 (70%)	116 (14%)
Vacuole	799	556 (70%)	79 (10%)
Cell wall	469	314 (67%)	45 (10%)
Plant-type cell wall	264	176 (67%)	26 (10%)
Endosome	232	153 (66%)	13 (6%)
Trans-Golgi network	219	144 (66%)	13 (6%)
Cytoplasm	3461	2222 (64%)	364 (11%)
Nucleus	9214	5924 (64%)	1118 (12%)
Extracellular region	2390	1512 (63%)	232 (10%)
Intracellular	1015	630 (62%)	148 (15%)
Cytosol	1468	903 (62%)	151 (10%)
Integral to membrane	808	503 (62%)	67 (8%)
Golgi apparatus	877	539 (61%)	65 (7%)
Plastid	289	172 (60%)	37 (13%)
Peroxisome	170	99 (58%)	17 (10%)
Mitochondrion	3048	1744 (57%)	323 (11%)
Cytosolic ribosome	304	164 (54%)	30 (10%)
Apoplast	390	208 (53%)	35 (9%)
Endoplasmic reticulum	517	270 (52%)	26 (5%)
Anchored to membrane	237	120 (51%)	16 (7%)
Ribosome	384	143 (37%)	33 (9%)
Cellular component	1917	705 (37%)	149 (8%)

Total number of proteins, number of predicted candidates for *S*-nitrosylation, and the number of candidates with the highest 10% prediction confidence were assigned to their subcellular localization according to gene ontology cellular component classification. The prediction confidence was calculated by dividing the raw score value by the cutoff value of a particular cluster.

The nucleus is an important sub-cellular organelle that contains almost all of the genetic information required for the regulation of cellular processes. Interestingly, a high number of S-nitrosylation candidates was predicted for the “nucleus” compartment (5,924 proteins, 64% of the total), which also contained a high proportion of the proteins that harbored the 10% of sites that were predicted with the highest confidence (1,118 proteins, 12% of the total).

The 10% of S-nitrosylation sites that were predicted with the highest confidence were also found in all compartments at levels of 5% to 17% (Table 3). In particular, the compartments “chloroplast” (17%), “intracellular” (15%), and “plasmodesmata” (14%) appeared to be enriched in the sites predicted with high confidence. Interestingly, chloroplast proteins exhibited the highest percentage of S-nitrosylation candidates in both analyses. Chloroplasts are sources of redox intermediates and chloroplast signaling pathways are triggered by the redox state of the plastochinone pool, the thioredoxin system, and the acceptor availability at photosystem I [56]. Moreover, discrete redox signaling pathways regulate photosynthetic light-harvesting and chloroplast gene transcription [57]. Production of NO in plant cells arise from several different pathways and in different organelles, including chloroplasts [58], [59] and target sites of NO in chloroplasts have been found in photosystem I and II, in the cytochrome *b*6*f* complex and in carbon dioxide reduction processes [60]. Although the chloroplast S-nitrosylome has not been analyzed yet, alterations in ribulose-1,5-bisphosphate carboxylase/oxygenase S-nitrosylation inactivated its carboxylase activity in *Brassica juncea* [61]. Furthermore, chloroplastic triosephosphate isomerase (TPI) was already identified as target for S-nitrosylation in rice, citrus, and *Chlamydomonas reinhardtii*, suggesting that this type of modification might be involved in the regulation of chloroplastic TPI activity [29], [30], [62], [63]. Moreover, chloroplasts have been discussed as a source and a target of cellular redox regulation [56] and therefore might represent a favorable microenvironment for S-nitrosylation in *Arabidopsis*.

In most compartments, the percentage of proteins predicted as S-nitrosylation candidates using the medium threshold ranged from 51% to 70%. The smallest proportion of S-nitrosylation candidates was located in the “ribosome” compartment (37%). Ribosomes comprise the basic machinery that decodes genetic information into proteins. Increasing numbers of studies on ribosome biogenesis have been performed on *Arabidopsis*. Ribosomal protein functions have been demonstrated in embryo biogenesis, leaf and flower development, vacuolar trafficking, and the UV response [64]–[70]. However, the lowest percentages of predicted targets among the 10% of sites predicted with the highest confidence were found in the “endoplasmic reticulum” (5%), “trans-Golgi network” (6%), and “endosome” (6%) compartments. Several previous studies have demonstrated that S-nitrosylated proteins were localized in various organelles, including the cell membrane [71], mitochondria [72], [73], the nucleus [74], the endoplasmic reticulum, the Golgi and cytosol [75], the peroxisome [31], and the apoplast [76]. This suggests that protein S-nitrosylation can occur in all subcellular compartments [77].

### 4. Functional distribution of *Arabidopsis* S-nitrosylation candidate proteins

**Figure 1:**
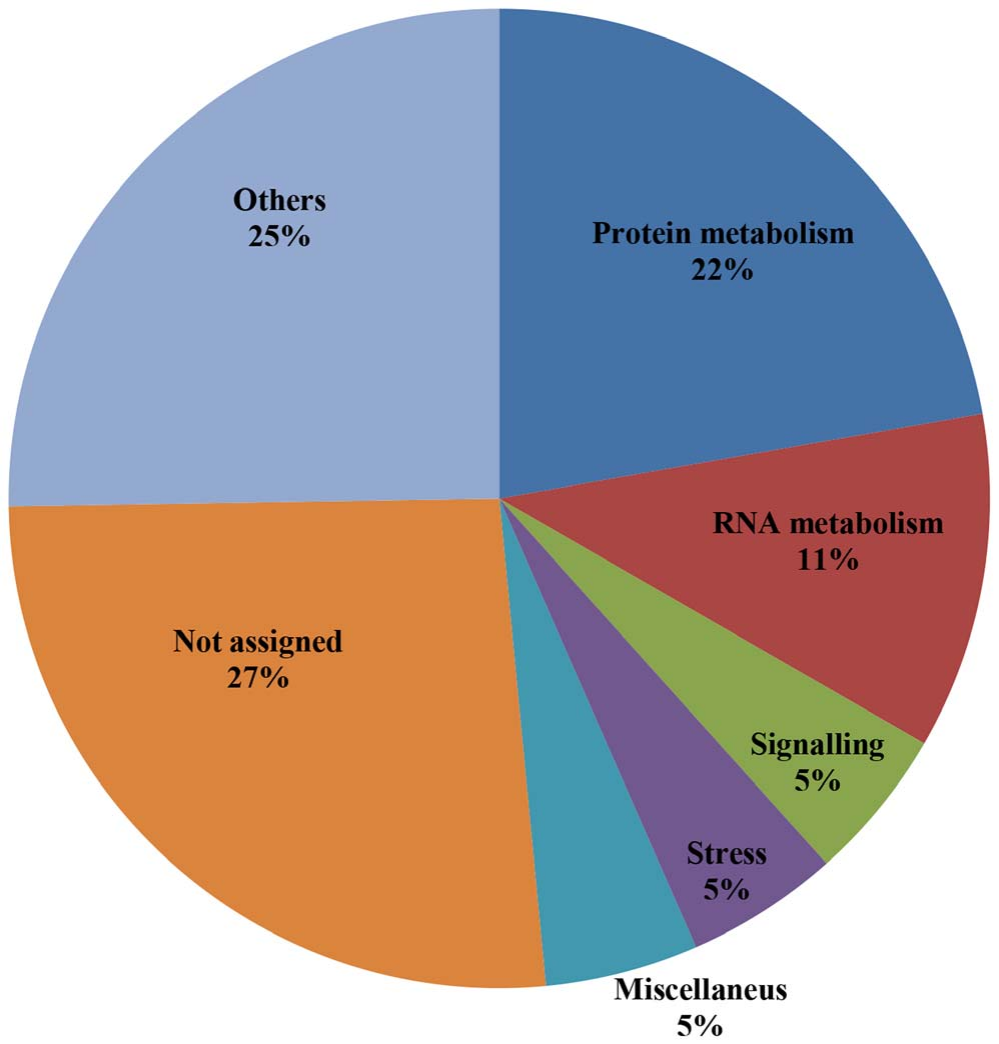
Functional distribution of predicted candidate proteins for *S*-nitrosylation has been determined using the MapMan Ontology tool (http://mapman.gabipd.org/). Others; include all functional classes which have less than 5% of predicted candidates.

**Figure 2:**
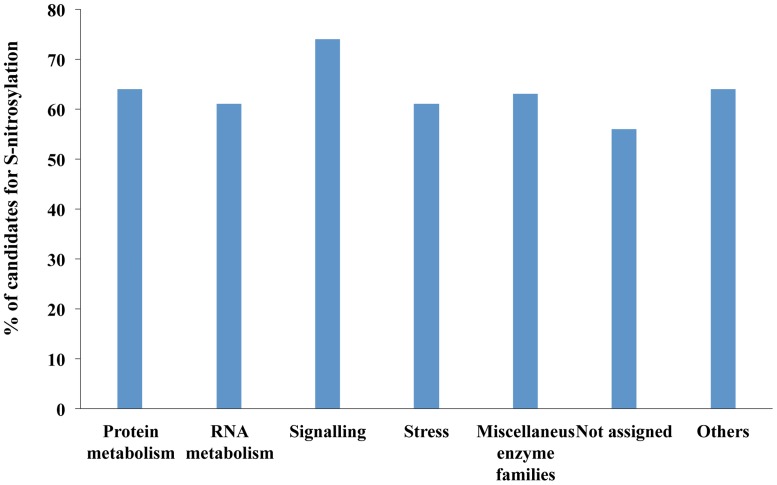
Percentage of candidate proteins for *S*-nitrosylation in different functional categories. Functional assignment has been done using the MapMan Ontology tool (http://mapman.gabipd.org/web/guest/mapman).

To analyze the functional classification of the predicted candidates, 16,610 predicted proteins were subjected to analysis using the MapMan Ontology of *Arabidopsis* proteins (http://mapman.gabipd.org/web/guest/mapman). Most of the candidates belong to unknown categories (not assigned) or others, including categories containing less than 5% of candidates (Figure 1). Most of the candidates assigned to known categories are involved in protein and RNA metabolism (22% and 11% of all candidates, respectively), signaling (5%) and stress-related processes (5%). The proportion of predicted candidates in known functional categories was calculated in relation to the total number of proteins of each category; the results showed that approximately 60% of the proteins in each category were S-nitrosylation candidates (Figure 2). The signaling category presented the highest proportion of S-nitrosylation candidates (70%). A more detailed analysis of this group revealed that 70% to 100% of the subclasses “14-3-3 family proteins”, “light”, “lipids”, “MAP and receptor kinases”, “phosphoinositides”, and “sugar and nutrient physiology”, are S-nitrosylation candidates (Table 4). 14-3-3 proteins have previously been identified as S-nitrosylation targets in *Arabidopsis* [16], [28] and in mesangial cells [78]. 14-3-3 proteins represent an emerging family of proteins and protein domains that bind to serine/threonine-phosphorylated residues. These proteins regulate key proteins that are involved in several physiological processes, including intracellular signaling, apoptosis, cell cycling, and transcriptional regulation. 14-3-3 proteins also act as adaptor molecules that stimulate protein-protein interactions and regulate the subcellular localization of proteins [79]. Interestingly, the 10% of sites predicted with the highest confidence in the large-scale prediction study showed the same functional classification pattern as that for all S-nitrosylated proteins (Figure S1). The functional distribution of the predicted S-nitrosylation candidates is similar to that of the major classes of S-nitrosylated proteins that have been identified experimentally in *Arabidopsis* [16], [27], [28], [80].

**Table 4:** Percentage of predicted candidate proteins for *S*-nitrosylation in signaling subclasses.

Signaling subclasses	Total proteins	Candidate proteins for *S*-nitrosylation
14-3-3 proteins	15	15 (100%)
Light	117	98 (84%)
Lipids	6	5 (83%)
MAP kinases	50	40 (80%)
Receptor kinases	1067	843 (79%)
Phosphinositides	98	76 (78%)
Sugar and nutrient physiology	82	58 (71%)
G-proteins	243	157 (65%)
Unspecified	8	5 (62%)
Calcium	230	141 (61%)
Miscellaneus enzyme families	41	21 (51%)
Phosphorelay	5	1 (20%)

Functional classification of the predicted candidates has been done using the MapMan Ontology software (http://mapman.gabipd.org/web/guest/mapman).

### 5. A comparison of experimentally identified candidates with the candidates predicted using GPS-SNO 1.0 software

Two-hundred sixty-three proteins have previously been identified experimentally in *Arabidopsis thaliana* as S-nitrosylation candidates based on the BST [16], [18], [19], [27], [28], [80]–[83]. These proteins were detected using large- and small-scale studies, most of which did not determine the exact S-nitrosylation sites experimentally. To compare the results of the computational predictions with experimental data, we analyzed these datasets using GPS-SNO. Interestingly, 160 proteins (approximately 61%) that were identified using the biotin switch approach were also predicted by the GPS-SNO software (using the medium threshold) as S-nitrosylation candidates.

In a more detailed analysis, Fares et al. experimentally identified 53 S-nitrosylation sites on 46 proteins in an *Arabidopsis* cell suspension using BS-ICAT technology [28]. However, these identified S-nitrosylation sites were not further verified on the biochemical and physiological level meaning that these S-nitrosylation sites/proteins are still candidates. This set of proteins was also analyzed using the GPS-SNO 1.0 software under the medium threshold condition (Table 5). This analysis revealed that approximately 74% of proteins (34 proteins) that were identified as S-nitrosylated using BS-ICAT were also predicted as S-nitrosylation candidates using GPS-SNO. To compare the candidate cysteine sites, the GPS-SNO program was used to predict 60 putative S-nitrosylation sites within these 34 proteins; however, only 11 of the predicted S-nitrosylation sites corresponded to sites identified using BS-ICAT (Table 6). These data indicate that the GPS-SNO software predicts a different set of S-nitrosylation sites in comparison to the BST-based approach.

**Table 5:** Prediction of *S*-nitrosylated sites from experimentally identified *S*-nitrosylated proteins by GPS-SNO software.

Accession number	Cys-NO site identified by BS-ICAT	Cys-NO site predicted by GPS-SNO	NO-peptide sequence predicted by GPS-SNO
AT1G04710	C_130_	C_184_	KFEQAHNCLLPMGIT
AT1G04710	-	C_363_	FASQFVYCRNKLGLD
AT1G04710	-	C_394_	LGATGARCVATLLHE
AT1G04710	-	C_417_	RFGVVSMCIGSGMGA
AT1G07890	C_32_	C_19_	YKKAVEKCRRKLRGL
AT1G07930	C_87_	C_111_	TGTSQADCAVLIIDS
AT1G09780	C_100_	C_355_	NGVSTFACSETVKFG
AT1G19570	C_20_	C_6_	**MALEICVKAAVGA
AT1G22300	C_98_	**C**_98_	EDELAKVCNDILSVI
AT1G35720	C_111_	**C**_111_	QVLMEVACTRTSTQL
AT1G47128	C_233_	C_161_	DQGGCGSCWAFSTIG
AT1G47128	C_342_	**C**_342_	IASSSGKCGIAIEPS
AT1G47128	-	C_200_	DTSYNEGCNGGLMDY
AT1G56070	C_370_	C_131_	GALVVVDCIEGVCVQ
AT1G56070	-	C_448_	ETVEDVPCGNTVAMV
AT1G60710	C_198_	C_5_	***MAEACGVRRMKL
AT1G60710	-	C_254_	KIVYEKVCAISEKKG
AT1G63000	C_162_	-	-
AT1G65930	C_75_	C_297_	LMTSVLVCPDGKTIE
AT1G65930	C_363_	**C**_363_	TEKLEAACVGTVESG
AT1G65930	C_269_	-	-
AT1G73010	C_98_	C_165_	GTCPPNMCKGLIIER
AT1G77120	C_243_	C_10_	TTGQIIRCKAAVAWE
AT1G77120	-	C_271_	GVDRSVECTGSVQAM
AT1G78830	C_374_	-	-
AT2G31390	C_298_	-	-
AT2G39730	C_175_	C_451_	NLPVPEGCTDPVAEN
AT2G44350	C_108_	C_210_	WEPTYEDCLNLIARV
AT2G45290	C_440_	**C**_440_	TRNLSQQCLNALAKA
AT2G45290	-	C_245_	EGISNEVCSLAGHWG
AT3G08580	C_130_	**C**_130_	PYKGIGDCFGRTIKD
AT3G09820	C_323_	-	-
AT3G09840	C_109_	C_425_	CTEAALQCIREKMDV
AT3G09840	-	C_575_	KARQSAPCVLFFDEL
AT3G11940	C_175_	C_69_	KRFRKAQCPIVERLT
AT3G17240	C_372_	-	-
AT3G47370	C_39_	-	-
AT3G51800	C_178_	-	-
AT3G53870	C_134_	C_97_	KVNNRGLCAIAQAES
AT3G55440	C_218_	C_13_	FVGGNWKCNGTAEEV
AT3G55440	C_127_	**C**_127_	QGLKVIACVGETLEE
AT3G56310	C_311_	C_117_	IHVNIDDCWSNLLRD
AT3G56310	-	C_422_	AQVDAHDCHMYVLTP
AT3G61440	C_72_	C_16_	LRRETIPCFSHTVRK
AT3G61440	-	C_87_	QEHFQPTCSIKDRPA
AT4G09320	C_43_	C_2_	******MCGLYINLF
AT4G09320	C_268_	-	-
AT4G11150	C_201_	C_121_	LKDLIVQCLLRLKEP
AT4G11150	-	C_134_	EPSVLLRCREEDLGL
AT4G11650	C_72_	-	-
AT4G13430	C_376_	C_12_	ISSSPFLCKSSSKSD
AT4G13940	C_244_	C_42_	EMPGLMACRTEFGPS
AT4G13940	C_268_	-	-
AT4G33030	C_357_	**C**_357_	DIRDTVQCVEIAIAN
AT4G33030	-	C_9_	AHLLSASCPSVISLS
AT5G02500	C_319_	**C**_319_	NMDLFRKCMEPVEKC
AT5G02500	-	C_326_	CMEPVEKCLRDAKMD
AT5G02500	-	C_609_	MKELESICNPIIAKM
AT5G14040	C_104_	C_194_	IIADIALCPFEAVKV
AT5G15490	C_350_	-	-
AT5G25100	C_104_	C_363_	YVGTGVQCLGMVLVT
AT5G44340	C_354_	**C**_354_	NNVKSSVCDIAPKGL
AT5G44340	-	C_12_	LHIQGGQCGNQIGAK
AT5G44340	-	C_238_	ATMSGVTCCLRFPGQ
AT5G61790	C_108_	-	-
AT5G62690	C_56_	C_12_	LHIQGGQCGNQIGAK
AT5G62690	C_301_	C_238_	ATMSGVTCCLRFPGQ
AT5G62690	-	C_354_	NNVKSTVCDIPPTGL
AT5G66760	-	C_4_	****MWRCVSRGFRA
AT5G66760	-	C_77_	EHGFNTACITKLFPT
AT5G66760	-	C_294_	TGIYGAGCLITEGSR
AT5G66760	-	C_457_	IVVFGRACANRVAEI
AT5G66760	C_526_	**C**_526_	QETLEEGCQLIDKAW
ATCG00340	C_559_	-	-
ATCG00490	C_192_	-	-
ATCG00490	C_427_	-	-

*S*-nitrosylated *Arabidopsis* candidate proteins published by Fares *et al*. (2011) were analysed by GPS-SNO software using the medium threshold condition.

C in bold, matched cysteine residues.

**Table 6:** Computational analysis of proteins, which *S*-nitrosylation sites were identified by BS-ICAT technology [28].

	BS-ICAT	GPS-SNO 1.0 medium threshold
**Protein number**	46	34
**Total number of Cys-NO**	53	60
**Matched Cys-NO with BS-ICAT**	-	11

## Conclusions

Protein S-nitrosylation has emerged as an important field of the study of post-translational modification and is increasingly studied in plants. However, the proteomic approaches used to identify proteins that are targets of S-nitrosylation are associated with a variety of technical difficulties, such as the existence of side reactions in multi-step procedures, the low abundance or instability of proteins, and instrumental inaccuracy. Computational methods can help to overcome these problems. Computational analyses can be performed easily on complex protein datasets obtained from databases, regardless of protein abundance or instability or the existence of complex chemical reactions. However, computational approaches also present disadvantages. Protein S-nitrosylation is an enzyme-independent chemical reaction that depends on many factors, all of which define whether a given cysteine residue will be sensitive to this modification. Although GPS-SNO 1.0 appears to predict S-nitrosylation sites with better accuracy, sensitivity, and specificity than other algorithms (Table S1), further research is required to improve the accuracy of the identification of S-nitrosylated sites. In this context, a set of non-SNO proteins would be helpful to calculate the sensitivity and specificity of the predictor.

Of greatest importance, all developed programs, including GPS-SNO 1.0, are based on the primary sequence of the studied proteins. However, the 3-dimensional (3D) structure of a protein also greatly affects its sensitivity to S-nitrosylation. The 3D structure defines which cysteine residues are accessible, and the amino acids surrounding a cysteine residue in the 3D structure determine the sensitivity of this residue to S-nitrosylation. Knowledge of the tertiary and quaternary structure of the protein may identify additional cysteines that might not be identified based on the primary sequence. Conversely, cysteine residues that are predicted to be S-nitrosylation targets might be excluded because they are inaccessible based on the spatial conformation. Therefore, knowledge of the high-resolution structure of the microenvironment around each cysteine residue is essential for defining the physicochemical features that determine S-nitrosylation specificity. Protein 3D structures have been already used to identify protein phosphorylation sites [84]. In that study linear motifs and spatial amino acid composition within a specific radial distance from the phosphorylated amino acid residue have been included [84]. But in general, computer-based prediction of S-nitrosylation candidates from *Arabidopsis* can offer a starting point for experimental verification and for further studies of S-nitrosylation in plants. The combination of computational prediction and experimental verification represents a good approach to better understand the molecular mechanisms and the regulatory functions of S-nitrosylation in plants. Nevertheless, both methods must be developed further to improve the precision with which S-nitrosylation targets are identified. Finally, the identified or predicted candidates must be confirmed using recombinant proteins, cysteine mutants and *in-vivo* approaches.

## Supporting Information

Figure S1 **Functional distribution of the 10% of candidates that were predicted with the highest confidence levels based on the MapMan Ontology of** ***Arabidopsis*** **proteins (** http://mapman.gabipd.org/web/guest/mapman **).** Others: functional classes with less than 5% of S-nitrosylated candidates.
(TIF)

Table S1 **Comparison of the performance of four software tools in predicting S-nitrosylation sites.** Accuracy, sensitivity and specificity were used to evaluate the performance.
(DOCX)

Table S2 **The** ***Arabidopsis*** **proteome was extracted from the TAIR 10 database, and proteins were assigned to cellular localizations according to the gene ontology cellular component classification.**
(XLS)

Table S3 **Amino acid sequences were downloaded for** ***Arabidopsis*** **from TAIR (** www.arabidopsis.org **) and analyzed using the GPS-SNO 1.0 program and the medium threshold.** The 10% of candidates that were predicted with the highest confidence were ranked by the raw score divided by the cutoff of a particular cluster.
(XLSX)

Table S4 **The** ***Arabidopsis*** **proteome was extracted from the TAIR 10 database, and proteins were assigned to cellular localizations according to the gene ontology cellular component classification.**
(XLS)

Text S1 **Subcellular compartments assigned according to the gene ontology cellular component classification (** http://amigo1.geneontology.org/cgi-bin/amigo/go.cgi **).**
(DOC)
