# Supplementary figures and images for: Computational Prediction of Candidate Proteins for S-Nitrosylation in Arabidopsis thaliana
Source: PLoS One. 2014 Oct 21;9(10):e110232. doi: 10.1371/journal.pone.0110232 (PMC4204854; doi:10.1371/journal.pone.0110232)

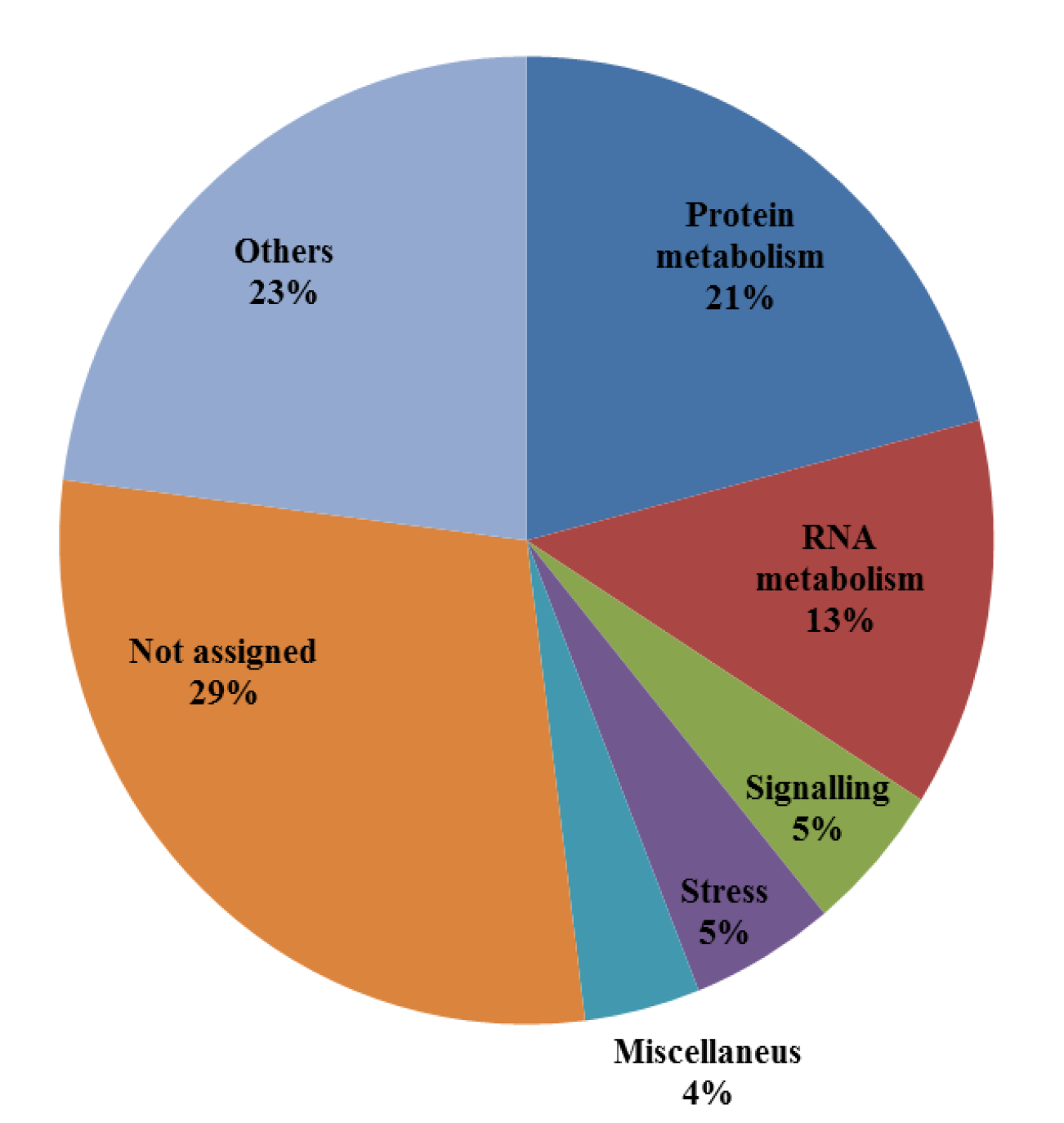

Supplement: Figure S1 — Functional distribution of the 10% of candidates that were predicted with the highest confidence levels based on the MapMan Ontology of Arabidopsis proteins ( http://mapman.gabipd.org/web/guest/mapman ). Others: functional classes with less than 5% of S-nitrosylated candidates. (TIF) [file pone.0110232.s001.tif]
