# Supplementary material for: Computational Prediction of Candidate Proteins for S-Nitrosylation in Arabidopsis thaliana
Source: PLoS One. 2014 Oct 21;9(10):e110232. doi: 10.1371/journal.pone.0110232 (PMC4204854; doi:10.1371/journal.pone.0110232)
Supplement: Table S1 — Comparison of the performance of four software tools in predicting S-nitrosylation sites. Accuracy, sensitivity and specificity were used to evaluate the performance. (DOCX) [file pone.0110232.s002.docx]

| **Software** | **Accuracy (%)** | **Sensitivity (%)** | **Specificity (%)** |
| --- | --- | --- | --- |
| GPS-SNO  (medium threshold) | 82,24 | 50,00 | 87,91 |
| iSNO-PseAAC | 57,94 | 37,50 | 61,54 |
| iSNO-AAPair | 67,29 | 18,75 | 75,82 |
| SNOSite | 25,23 | 93,75 | 13,19 |

**Table S1.** Comparison of GPS-SNO with other algorithms.
