# Supplementary material for: Computational Prediction of Candidate Proteins for S-Nitrosylation in Arabidopsis thaliana
Source: PLoS One. 2014 Oct 21;9(10):e110232. doi: 10.1371/journal.pone.0110232 (PMC4204854; doi:10.1371/journal.pone.0110232)
Supplement: Text S1 — Subcellular compartments assigned according to the gene ontology cellular component classification ( http://amigo1.geneontology.org/cgi-bin/amigo/go.cgi ). (DOC) [file pone.0110232.s006.doc]

**Supplemental Material S1.** Subcellular compartments according to gene ontology cellular component classification (http://amigo1.geneontology.org/cgi-bin/amigo/go.cgi)

C**hloroplast:** contains three main compartments. The inner-membrane system (thylakoids) essentially marks the site of light-dependent reactions of photosynthesis. These thylakoids are embedded in an intrachloroplast space (the stroma) that hosts many essential metabolic processes, including CO_2_ fixation. The envelope mainly controls exchanges between the chloroplast and the other plant cell compartments.

**CUL4-RING ubiquitin ligase complex:** A ubiquitin ligase complex in which a cullin from the Cul4 family and a RING domain protein form the catalytic core; substrate specificity is conferred by an adaptor protein.

**Membrane:** Double layer of lipid molecules that encloses all cells, and, in eukaryotes, many organelles; may be a single or double lipid bilayer; also includes associated proteins. In this compartment we included also the plasma membrane proteins.

**Plasmodesmata:** A fine cytoplasmic channel, found in all higher plants, that connects the cytoplasm of one cell to that of an adjacent cell.

**Vacuole:** A closed structure, found only in eukaryotic cells, that is completely surrounded by unit membrane and contains liquid material. Cells contain one or several vacuoles, that may have different functions from each other. Vacuoles have a diverse array of functions. They can act as a storage organelle for nutrients or waste products, as a degradative compartment, as a cost-effective way of increasing cell size, and as a homeostatic regulator controlling both turgor pressure and pH of the cytosol. Vacuolar membrane proteins were included in this compartment.

**Cell wall:** The rigid or semi-rigid envelope lying outside the cell membrane of plant, fungal, most prokaryotic cells and some protozoan parasites, maintaining their shape and protecting them from osmotic lysis. In plants it is made of cellulose and, often, lignin; in fungi it is composed largely of polysaccharides; in bacteria it is composed of peptidoglycan; in protozoan parasites such as Giardia species, it's made of carbohydrates and proteins.

**plant-type cell wall:** A more or less rigid structure lying outside the cell membrane of a cell and composed of cellulose and pectin and other organic and inorganic substances.

**Endosome:** Is a membrane-bounded compartment inside eukaryotic cells. It is a compartment of the endocytic membrane transport pathway from the plasma membrane to the lysosome.

**Trans-Golgi network:** The network of interconnected tubular and cisternal structures located within the Golgi apparatus on the side distal to the endoplasmic reticulum, from which secretory vesicles emerge. The trans-Golgi network is important in the later stages of protein secretion where it is thought to play a key role in the sorting and targeting of secreted proteins to the correct destination. trans-Golgi network is not considered part of the Golgi apparatus but is a separate organelle.

**Cytoplasm:** All of the contents of a cell excluding the plasma membrane and nucleus, but including other subcellular structures.

**Nucleus:** A membrane-bounded organelle of eukaryotic cells in which chromosomes are housed and replicated. In most cells, the nucleus contains all of the cell's chromosomes except the organellar chromosomes, and is the site of RNA synthesis and processing. In some species, or in specialized cell types, RNA metabolism or DNA replication may be absent. Nucleolus proteins were included in this compartment.

**Extracellular region:** Is the space external to the outermost structure of a cell. For cells without external protective or external encapsulating structures this refers to space outside of the plasma membrane. This term covers the host cell environment outside an intracellular parasite.

**Intracellular: synonym** protoplasm: The living contents of a cell; the matter contained within (but not including) the plasma membrane, usually taken to exclude large vacuoles and masses of secretory or ingested material. In eukaryotes it includes the nucleus and cytoplasm.

**Cytosol:** The part of the cytoplasm that does not contain organelles but which does contain other particulate matter, such as protein complexes.

**Integral to membrane: synonym** transmembrane: Penetrating at least one phospholipid bilayer of a membrane. May also refer to the state of being buried in the bilayer with no exposure outside the bilayer. When used to describe a protein, indicates that all or part of the peptide sequence is embedded in the membrane.

**Golgi apparatus:** A compound membranous cytoplasmic organelle of eukaryotic cells, consisting of flattened, ribosome-free vesicles arranged in a more or less regular stack. The Golgi apparatus differs from the endoplasmic reticulum in often having slightly thicker membranes, appearing in sections as a characteristic shallow semicircle so that the convex side (cis or entry face) abuts the endoplasmic reticulum, secretory vesicles emerging from the concave side (trans or exit face). In vertebrate cells there is usually one such organelle, while in invertebrates and plants, where they are known usually as dictyosomes, there may be several scattered in the cytoplasm. The Golgi apparatus processes proteins produced on the ribosomes of the rough endoplasmic reticulum; such processing includes modification of the core oligosaccharides of glycoproteins, and the sorting and packaging of proteins for transport to a variety of cellular locations. Three different regions of the Golgi are now recognized both in terms of structure and function: cis, in the vicinity of the cis face, trans, in the vicinity of the trans face, and medial, lying between the cis and trans regions.

**Plastid:** Any member of a family of organelles found in the cytoplasm of plants and some protists, which are membrane-bounded and contain DNA. Plant plastids develop from a common type, the proplastid.

**Peroxisome:** A small organelle enclosed by a single membrane, and found in most eukaryotic cells. Contains peroxidases and other enzymes involved in a variety of metabolic processes including free radical detoxification, lipid catabolism and biosynthesis, and hydrogen peroxide metabolism.

**Mitochondrion:** A semiautonomous, self replicating organelle that occurs in varying numbers, shapes, and sizes in the cytoplasm of virtually all eukaryotic cells. It is notably the site of tissue respiration.

**Cytosolic ribosome:** A ribosome located in the cytosol.

**Apoplast:** The cell membranes and intracellular regions in a plant are connected through plasmodesmata, and plants may be described as having two major compartments: the living symplast and the non-living apoplast. The apoplast is external to the plasma membrane and includes cell walls, intercellular spaces and the lumen of dead structures such as xylem vessels. Water and solutes pass freely through it.

**Endoplasmic reticulum:** The irregular network of unit membranes, visible only by electron microscopy, that occurs in the cytoplasm of many eukaryotic cells. The membranes form a complex meshwork of tubular channels, which are often expanded into slitlike cavities called cisternae. The endoplasmic reticulum takes two forms, rough (or granular), with ribosomes adhering to the outer surface, and smooth (with no ribosomes attached).

**Anchored to membrane**: The component of a membrane consisting of the gene products that are tethered to the membrane only by a covalently attached anchor, such as a lipid group that is embedded in the membrane. Gene products with peptide sequences that are embedded in the membrane are excluded from this grouping.

**Ribosome:** An intracellular organelle, consisting of RNA and protein. It is the site of protein biosynthesis resulting from translation of messenger RNA (mRNA). It consists of two subunits, one large and one small, each containing only protein and RNA. Both the ribosome and its subunits are characterized by their sedimentation coefficients, expressed in Svedberg units (symbol: S). Hence, the prokaryotic ribosome (70S) comprises a large (50S) subunit and a small (30S) subunit, while the eukaryotic ribosome (80S) comprises a large (60S) subunit and a small (40S) subunit. Two sites on the ribosomal large subunit are involved in translation, namely the aminoacyl site (A site) and peptidyl site (P site). Ribosomes from prokaryotes, eukaryotes, mitochondria, and chloroplasts have characteristically distinct ribosomal proteins.

**Cellular component:** The part of a cell or its extracellular environment in which a gene product is located. A gene product may be located in one or more parts of a cell and its location may be as specific as a particular macromolecular complex, that is, a stable, persistent association of macromolecules that function together. This term is recommended for use for the annotation of gene products whose cellular component is unknown.
